# Evolution and Epidemic Spread of the Avian Infectious Bronchitis Virus (IBV) GI-23 in Brazil

**DOI:** 10.3390/v15061229

**Published:** 2023-05-24

**Authors:** Nilo Ikuta, Diéssy Kipper, Dayana Soriano Spencer de Freitas, André Salvador Kazantzi Fonseca, Vagner Ricardo Lunge

**Affiliations:** 1 Simbios Biotecnologia, Cachoeirinha 94940-030, RS, Brazil; ikuta@simbios.com.br (N.I.); diessykipper@hotmail.com (D.K.); fonseca@simbios.com.br (A.S.K.F.); 2 Laboratory of Molecular Diagnostic, Lutheran University of Brazil (ULBRA), Canoas 92425-900, RS, Brazil; dayspencerfreitas@rede.ulbra.br; 3 Institute of Biotechnology, University of Caxias do Sul (UCS), Caxias do Sul 95070-560, RS, Brazil

**Keywords:** avian, IBV, HVR1/2, phylodynamic

## Abstract

Infectious bronchitis virus (IBV) is a pathogen affecting poultry flocks worldwide. GI-23 is an IBV lineage with a rapid spread into different continents of the world, and it was reported for the first time in South American/Brazilian broiler farms last year. This study aimed to investigate the recent introduction and epidemic spread of IBV GI-23 in Brazil. Ninety-four broiler flocks infected with this lineage were evaluated from October 2021 to January 2023. IBV GI-23 was detected using real-time RT-qPCR, and the S1 gene hypervariable regions 1 and 2 (HVR1/2) were sequenced. S1 complete and HVR1/2 nucleotide sequence datasets were used to carry out phylogenetic and phylodynamic analyses. Brazilian IBV GI-23 strains clustered into two specific subclades (SA.1 and SA.2), both in tree branches with IBV GI-23 from Eastern European poultry-producing countries, suggesting two independent and recent introductions (around 2018). Viral phylodynamic analysis showed that the IBV GI-23 population increased from 2020 to 2021, remaining constant for one year and declining in 2022. S1 amino acid sequences from Brazilian IBV GI-23 presented specific and characteristic substitutions in the HVR1/2 for subclades IBV GI-23 SA.1 and SA.2. This study brings new insights into the introduction and recent epidemiology of IBV GI-23 in Brazil.

## 1. Introduction

Infectious bronchitis virus (IBV) is a pathogen of domestic fowl (*Gallus gallus*), causing severe respiratory, renal and reproductive disorders in commercial poultry flocks worldwide [1]. It is a *Gammacoronavirus* from the family *Coronaviridae* and presents a high genetic heterogeneity, having previously been divided into 6 genetic types (GI-GVI) and 32 (1–32) lineages spread worldwide by two comprehensive classification studies [2,3].

The IBV genome is a single positive-sense RNA strand about 27.6 Kb in length encoding the RNA-dependent RNA polymerase as well as the structural nucleocapsid (N), membrane (M), envelope (E) and spike (S) proteins. As with other coronaviruses, S is separated into the subunits S1 and S2 with approximately 535 and 625 amino acids, respectively. S1 glycoprotein is necessary for adsorption to the cellular receptor and S2 is critical for virus entry into the host cell. The S1 gene is highly diverse among different viral lineages/strains, highlighting three hypervariable regions (HVR1/2/3) along the nucleotide sequences. S1 protein is also directly related to IBV antigenicity and the induction of neutralizing antibodies in the host [4,5].

IBV genetic type I lineage 23 (GI-23) emerged in the Middle East and has been increasingly detected in severe respiratory infections from poultry flocks worldwide. This lineage (also called Variant 2) has reached Europe and Africa in the last few decades and was first reported in Brazil last year [6,7]. Due to the huge economic losses to the poultry industry, IBV GI-23-specific vaccination programs have been used in commercial farms [8,9]. The present study aimed to describe the recent introduction and epidemic spread of IBV GI-23 in Brazilian broiler flocks.

## 2. Materials and Methods

### 2.1. Sample Collection

Dead birds from 94 broiler flocks with animals presenting suggestive clinical signs of IBV GI-23 infection were necropsied in loco in South and Southeast Brazil from October 2021 to January 2023 (Table S1). Chicken tissues and organs (trachea, lungs, kidneys, cecal tonsils) were separated and packed in plastic bags, transported in ice boxes and maintained at −20 °C in a freezer until laboratory analyses.

### 2.2. Molecular Biology Methods

Cotton-tipped swabs were scraped against all chicken tissues and organs. Total nucleic acid was extracted from these samples with commercial reagents NewGene (Preamp and Prep) according to the supplier instructions (Simbios Biotecnologia, Cachoeirinha, Brazil). IBV was detected using reverse transcription and real-time TaqMan^®^ polymerase chain reaction (RT-qPCR) targeting the 5′ untranslated region (5′UTR) with the commercial reagent NewGene IBVAmp (Simbios Biotecnologia, Cachoeirinha, Brazil). All samples were also evaluated with three other RT-qPCRs (targeting specific S1 IBV gene regions) for the specific detection of IBV lineages GI-1 (Massachusetts), GI-11 (Brazilian field variants) and GI-23 (Variant 2) with the commercial reagent NewGene (Simbios Biotecnologia, Cachoeirinha, Brazil). All RT-qPCR assays were carried out in a StepOnePlusTM Real-Time PCR System (Applied Biosystems, Norwalk, CT, USA) with the following conditions: 1 cycle of 37 °C for 30 min, followed by 40 cycles at 95 °C for 15 s and 60 °C for 1 min.

Nucleotide sequencing of S1 HVR1/2 was carried out using a nested RT-PCR as previously reported [7,10]. Briefly, the RT-PCR was prepared in a total volume of 25 µL, using 2 µL of RNA template, 16 µL of H_2_O, 0.6 µL of dithiothreitol (0.1 M), 0.7 µL of deoxynucleotide triphosphate (2.1 mM of deoxyadenosine triphosphate, deoxythymidine triphosphate, deoxycytidine triphosphate and deoxyguanosine triphosphate), 0.2 µL of the primers S15 and CK2 (50 mM each), 0.1 µL of RNAguard 40 U/mL, 0.1 µL of MMLV reverse transcriptase 200 U/mL (Promega, Madison, WI, USA), 0.2 µL of Taq DNA polymerase (5 U/mL) and 5 µL of a concentrate RT and Taq DNA polymerase buffer (Ludwig Biotech, Alvorada, Brazil). Amplification was carried out in a thermocycler Veriti 96 (Applied Biosystems, Waltham, MA, USA) with the following conditions: 1 cycle at 37 °C for 30 min and 35 cycles at 94 °C for 20 s, 50 °C for 40 s and 72 °C for 1 min. The nested PCR was also prepared in a volume of 25 µL using 2 µL of RT-PCR-amplified DNA, 18.5 µL of treated H_2_O, 0.8 µL of MgCl2 (50 mM), 0.7 µL of deoxyribonucleotides triphosphates (2.1 mM), 0.2 µL of primers IBVS1-1F and IBVS1-3R (50 mM each), 0.3 µL of Taq DNA polymerase (5 U/mL) and 2.5 µL of Taq DNA polymerase buffer (Ludwig Biotech, Alvorada, Brazil). Thermal cycling was carried out in the same thermocycler with the following steps: 1 cycle at 94 °C for 3 min, 35 cycles at 94 °C for 20 s, 55 °C for 40 s and 72 °C for 1 min, and a final extension cycle at 72 °C for 5 min. Nested RT-PCR-amplified 590 bp products covering the HVR1/2 S1 gene region were evaluated on polyacrylamide gel electrophoresis stained with silver nitrate.

PCR-amplified products were purified using the NewGene^®^ PCR Purification Kit in accordance with the manufacturer’s instructions (Simbios Biotecnologia, Cachoeirinha, Brazil). These products were sequenced using sense/antisense nested PCR primers (IBVS1-1F and IBVS1-3R) and a BigDye Terminator v. 3.1 Cycle Sequencing Kit (Applied Biosystems, Waltham, MA, USA). The sequencing rounds were performed in the thermocycler Veriti 96 (Applied Biosystems, Waltham, MA, USA) with the same conditions as above. All samples were purified through ethanol–EDTA–sodium acetate precipitation, and the precipitated DNA products were diluted in formamide Hi-Di, denatured (95 °C for 2 min) and injected in the automated DNA sequencing ABI 3500 XL Genetic Analyzer (Applied Biosystems, Waltham, MA, USA). Nucleotide sequences were obtained using Data Collection v. 1.0.1 (Applied Biosystems Inc.), and electropherograms were analyzed with Sequencing Analysis v. 5.3.1. software (Applied Biosystems, Waltham, MA, USA). Nucleotide sequences from both strands were edited, assembled and analyzed using Geneious software v. 2021.2.2 (Biomatters, Newark, NJ, USA). 

### 2.3. GenBank DATA Collection

IBV GI-23 S1 complete gene and HVR1/2 partial sequences with the collection date and country available in GenBank were downloaded and compared with two previously constructed reference datasets [7,11]. HVR1/2 sequences were extracted from nucleotide positions 112 to 423 of the S1 subunit complete gene, respectively, according to the sequence M21883 [2].

A total of 232 S1 complete gene sequences and 389 HVR1/2 sequences (including the 94 HVR1/2 IBV GI-23 strains sequenced in this study) were included in the two datasets, representing 9 and 13 countries, respectively (Tables S2 and S3). Furthermore, a total of 120 HVR1/2 sequences from Brazilian IBV GI-23 strains, including the 94 data sequenced in this study, were used in a separate dataset to perform temporal analysis (Table S4). All datasets were aligned using MAFFT v. 7 [12].

Phylogenetic trees were reconstructed using the maximum likelihood (ML) method implemented in the W-IQ-TREE web server [13]. The optimal nucleotide substitution model was selected using ModelFinder [14], and 1000 replicates of the ultrafast bootstrap approximation were used [15].

### 2.4. Recombination Analysis

Recombination events were verified using RDP5 v. 5.34 [16] with the default settings using the algorithms RDP, GENECONV, BootScan, MaxChi, Chimaera, SiScan, 3Seq and LARD. The beginning and end breakpoints of the potential recombinant sequences were also defined using the RDP5 software. Putative recombinant events were considered significant when *p* ≤ 0.01 was observed for the same event using four or more algorithms.

### 2.5. Phylodynamic Analysis

The linear regression approach implemented in TempEst v. 1.5 [17] was used to evaluate the temporal signal and clock-likeness of the ML phylogeny constructed using the three IBV GI-23 datasets: S1 complete gene, HVR1/2 global and HVR1/2 Brazilian. The resulting R2 values produced by TempEst were 0.13, 0.22 and 0.25, respectively (the best-fitting roots were used).

Non-recombinant datasets alignments were analyzed using the Bayesian serial coalescent approach implemented in BEAST v. 1.8.2 [18]. The nucleotide substitution model (GTR + G) was selected based on the Bayesian information criterion calculated using JModelTest v. 2.1.10 [19], while the relaxed lognormal molecular clock and the Bayesian coalescent skyline were selected. For each dataset, three independent runs of 350 million generations were performed. The resulting log files were viewed in Tracer v. 1.6 [20] to ensure that ESS values were sufficiently high (i.e., ≥200 for all parameters) and that all parameters had mixed adequately with 10% burn-in. LogCombiner v. 1.8.3 was used to combine the log and tree files of five independent runs, and TreeAnnotator v. 1.8.2 [21] was used to construct a maximum clade credibility (MCC) tree, using 10% burn-in and common ancestor node heights. FigTree v. 1.4.2 was used to annotate the resulting phylogeny, using bars to denote 95% highest posterior density (HPD) intervals for node heights and branch labels to denote posterior probabilities (http://tree.bio.ed.ac.uk/software/figtree/, accessed on 1 February 2023).

### 2.6. Amino Acid Substitutions Analysis

Alignments of the HVR1/2 amino acids (from residues 38 to 141) of the S1 gene [2] were generated with MAFFT v. 7 [12]. The main amino acid substitutions were visually inspected in Geneious v. 2021.2.2 (Biomatters, Newark, NJ, USA). The Israeli strain AF095796 was used as a reference in the comparison with the 120 IBV GI-23 strains from Brazil.

## 3. Results

### 3.1. IBV GI-23 Molecular Detection

IBV RNA was detected in all nucleic acid samples extracted from the tissues and organs of chickens from 94 different broiler flocks. All 94 RNA samples also presented positive results in the RT-qPCRs for IBV GI-23 and negative results in the other two IBV-lineage-specific assays (to detect IBV GI-1 and G-11). These same samples could be successfully amplified using the nested RT-PCR for the specific HVR1/2 genetic regions inside the S1 gene, and they were further sequenced, resulting in the data published in GenBank with the numbers OQ884498–OQ884591 (Table S1).

### 3.2. Recombination Analyses

The complete S1 gene and partial HVR1/2 datasets were subjected to recombination analyses. Twenty-eight recombination events in 32 IBV GI-23 S1 complete gene sequences were detected using RDP5 (Table S2). On the contrary, only two recombination events in five HVR1/2 sequences were observed using this same procedure (Table S3). All sequences with recombination events were removed, resulting in 200 S1 complete gene and 384 HVR1/2 sequences in these two IBV GI-23 datasets, respectively. No recombination event was identified in the dataset with 120 HVR1/2 IBV GI-23 Brazilian sequences (Table S4).

### 3.3. Phylogenetic Analyses

The dataset with the 200 IBV GI-23 S1 complete sequences from Brazil and other countries was used to construct a preliminary phylogenetic and temporal tree. The IBV GI-23 Brazilian reference sequence (SB-2805, 2021) clustered together with other IBV GI-23 sequences from Israel (2006), Poland (2015 to 2020), Romania (2016 to 2020) and Turkey (2014 to 2019) in a very characteristic and independent branch. A first estimate showed that SB-2805 was probably introduced into Brazil between 2018 and 2019 (Figure S1).

**Figure 1:**
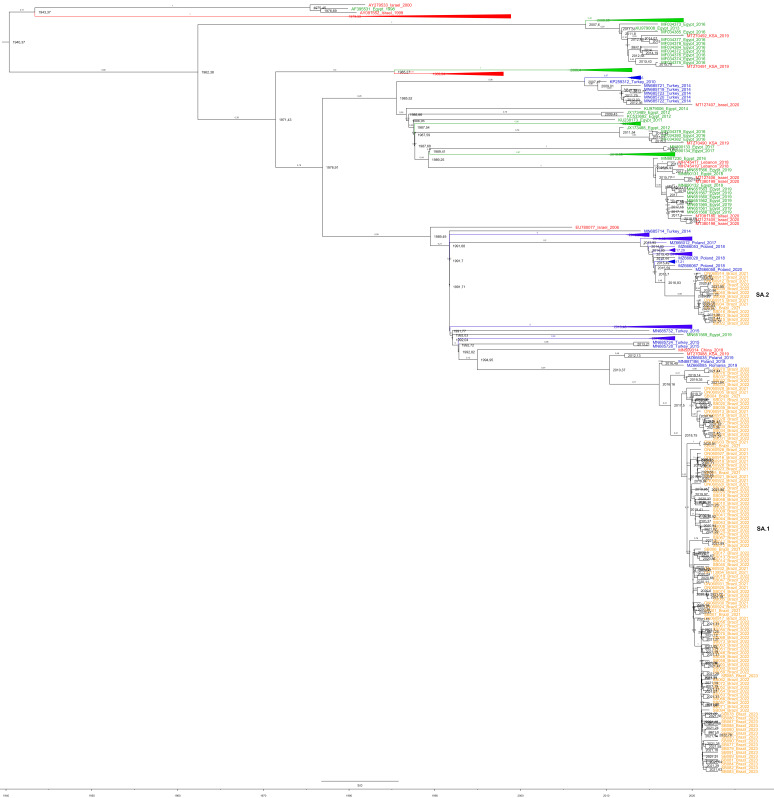
Maximum clade credibility tree constructed using 384 HVR1/2 sequences from around the world, rooted using BEAST. Tip label colors denote the geographic origin of isolates (green for Africa, red for Asia, blue for Europe and purple for South America, with internal clades collapsed). Time by year is plotted along the x-axis. Branch labels denote posterior probabilities of branch support.

The dataset with 384 HVR1/2 IBV GI-23 sequences was also evaluated. Again, an independent branch with all Brazilian, one Israel and several European (from Poland, Romania and Turkey) IBV GI-23 sequences was also observed. However, this clade also included sequences from China, South Africa (KSA) and Egypt. Importantly, Brazilian IBV GI-23 could be clearly separated into two subclades (SA.1 and SA.2), suggesting at least two different viral ancestors (Figure 1).

### 3.4. Phylodynamic Analyses

The first phylodynamic analysis was performed with the dataset including 384 HVR1/2 IBV GI-23 sequences, including IBV strains from Brazil (n = 120), China (n = 1), Egypt (n = 45), Germany (n = 1), Iran (n = 14), Iraq (n = 5), Israel (n = 15), Saudi Arabia (n = 4), Lebanon (n = 2), Nigeria (n = 2), Poland (n = 102), Romania (n = 12) and Turkey (n = 61), obtained from 1996 to 2023 (Table S3). The phylodynamic analysis showed that 384 HVR1/2 sequences from around the world presented a tMRCA (time to the most recent common ancestor) dating from 1940. In this set, the Brazilian IBV GI-23 sequences grouped into two subclades: Group 1 (SA.1), including 106 Brazilian sequences with a tMRCA dating around 2017; Group 2 (SA.2), including 14 Brazilian sequences with a tMRCA dating around 2020 (Figure 1). The evolutionary rate for the set of 384 HVR1/2 sequences from around the world was predicted to be 4.3 × 10^−3^ replacements/site/year (95% HPD = (2.6 × 10^−3^) to (5.7 × 10^−3^)). 

**Figure 2:**
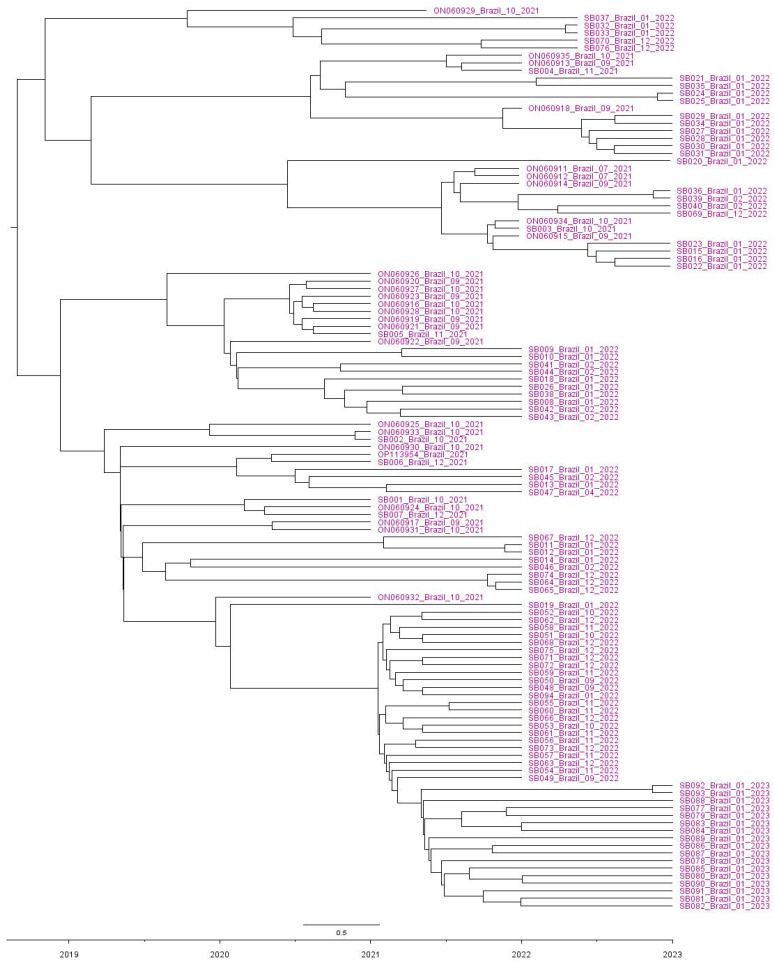
Maximum clade credibility tree constructed using 120 HVR1/2 Brazil sequences, rooted using BEAST. Tip label colors denote the geographic origin of isolates (purple for South America). Time by year is plotted along the x-axis. Branch labels denote posterior probabilities of branch support.

**Figure 3:**
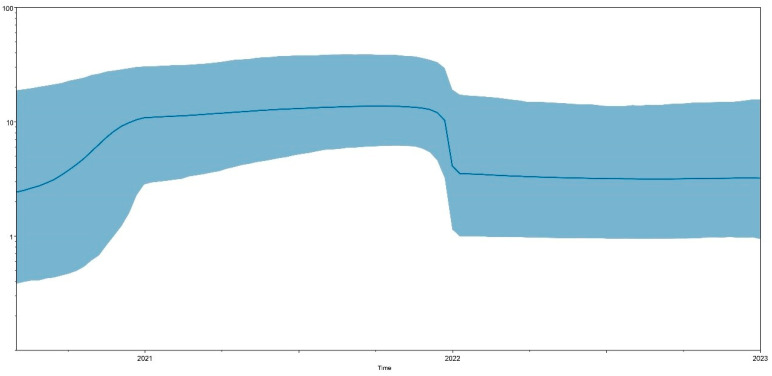
Bayesian skyline plot constructed via BEAST and Tracer using 120 HVR1/2 Brazil sequences. The x-axis represents time by year, while the y-axis denotes the effective population size. The dark-blue line represents the median, while light-blue shading represents the 95% HPD interval.

Furthermore, all 120 HVR1/2 sequences from Brazil showed that the tMRCA dates to around 2018 (Figure 2). The evolutionary rate for these 120 Brazilian HVR1/2 sequences was predicted to be 5.1 × 10^−3^ replacements/site/year (95% HPD = (1.9 × 10^−3^) to (8.6 × 10^−3^)). The Bayesian skyline assessment showed that the population of 120 Brazilian HVR1/2 sequences underwent an increase between July 2020 and January 2021. From January 2021 to approximately December 2021, it remained at a constant high. From December 2021 to January 2022, it suffered a drop until the current year (Figure 3).

### 3.5. S1 HVR1/2 Amino Acid Substitutions

A visual inspection of amino acid substitutions in the HVR1/2 region of the S1 gene (from amino acid 38 to 141) was performed comparing the Israeli reference strain AF095796 with the 120 IBV GI-23 genetic sequences from Brazil. 

**Figure 4:**
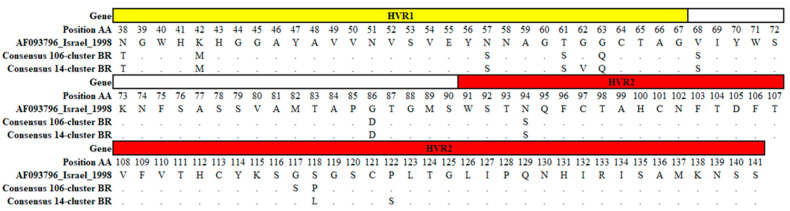
Schematic representation of amino acid substitutions present in AF093796, consensus 106-cluster BR (SA.1) and consensus 14-cluster BR (SA.2) in HVR1 (yellow) and HVR2 (red) region. Amino acid changes are represented with a different letter and those that were conserved are indicated with dots.

Eight amino acid changes between AF095796 and all IBV GI-23 Brazilian sequences (including SA.1 and SA.2 subclades) were identified: N38T, K42M, N57S, T61S, G63Q, V68S, G86D and N94S. Furthermore, there were only two amino acid substitutions in the IBV GI-23 subclade SA.1 (G117S and S118P) and three amino acid substitutions in the subclade SA.2 (G62V, S118L, P122S) (Figure 4).

## 4. Discussion

IBV is an avian pathogen presenting very high genetic heterogeneity, with many new genetic types and lineages being frequently identified worldwide [22,23]. Among them, IBV GI-23 is a very concerning lineage causing severe infectious bronchitis, having spread significantly in commercial poultry worldwide since the 2000s. It was very recently reported in Brazil, being associated with huge economic losses to the broiler industry in the southern poultry-producing regions in the last two years [7]. To better understand the current epidemic, the main aims of this study were to determine the introduction time and geographic origin of this novel IBV lineage spreading into Brazil as well as to evaluate several specific S1 structural properties of Brazilian strains. 

Therefore, a total of 94 IBV GI-23 S1 partial gene sequence data were obtained from different strains (detected in different broiler flocks with IBV) in Brazil from 2021 and 2023. More complete IBV GI-23 nucleotide sequences datasets (with the S1 gene and partial HVR1/2 regions) were then constructed to obtain trustable phylogenetic and evolutionary analyses. Although S1 complete gene sequencing has been proposed for robust phylogenetic evaluations, partial HVR1/2 has already been demonstrated to be useful for IBV GI-23 evolutionary analyses [11]. Additionally, an additional procedure was performed to detect recombinant IBV GI-23 sequences. Noteworthily, there were only two events in five recombinant HVR1/2 sequences, all of which were removed from the remaining analyses. As largely demonstrated by IBV and other coronaviruses, recombination events can occur frequently. However, most of these genetic events generate unique variants (UVs) or recombinant forms (URFs) with very restricted circulation in poultry flocks [24].

The analysis of complete S1 gene sequences demonstrated that the unique Brazilian IBV GI-23 clustered with Eastern European IBV sequences from this same lineage. In the phylogenetic evaluation of the HVR1/2 partial S1 sequences, Brazilian strains could be classified into two clearly separated subclades: (i) SA.1, with 106 sequences and (ii) SA.2, with 14 sequences. Interestingly, both IBV GI-23 Brazilian subclades clustered with other sequences from Poland, Romania and Turkey, suggesting Eastern Europe is the origin of these local IBV variants. The introduction period into Brazil was also estimated, and the results demonstrate that IBV GI-23 probably arrived in Brazilian poultry farms between 2017 and 2019. The phylodynamic analysis reinforced the occurrence of two Brazilian IBV GI-23 subclades (SA.1 and SA.2), highlighting that more than one introduction occurred in this time period. Other avian viruses (such as infectious bursal disease virus, IBDV) have also probably been introduced into Brazil from Europe: vvIBDV (originating from The Netherlands) and avIBDV G4 (originating from Eastern Europe) [25,26]. 

The reasons behind the likely spread of IBV GI-23 from Europe to South America could not be definitively answered here. However, we can raise some hypotheses. Brazil is a very important poultry and other-livestock producer and trader in the world. A marked growth in poultry flocks for chicken and egg production has been observed in the country since the beginning of the 2000s, thus resulting in increased international live-poultry trade [27,28]. Despite the rigorous biosecurity measures in place in Brazil to prevent poultry pathogens’ dissemination across borders, very few imports of embryonated eggs and live birds could have been responsible for the introduction of IBV GI-23 into the country. It is also necessary to consider that fomites (feed/slaughter contacts) and human movements (company technician, veterinarian, shared farm workers) could be linked to this recent introduction of IBV GI-23. All these possibilities were already associated with the introduction of avian influenza virus (AIV) into different poultry farms and geographic regions [29,30]. There is a strong positive relationship between the opening of new markets and the introduction of a range of animal diseases, as well as a strong association of the growing trade volumes with the probability that poultry viral diseases will establish and spread [30,31]. 

After its introduction, IBV GI-23 rapidly spread through high-poultry-density farms in the south and southeast regions of the country. Detailed knowledge of how poultry farms are connected to each other can help contain IBV GI-23. The disruption of the potential transmission network between poultry farms is necessary to control the continued spread of viral diseases, as previously demonstrated [30]. This is necessary for IBV as well as the highly pathogenic AIV recently detected in South America [32]. 

Until recently, GI-1, GI-11 and GI-16 were the only lineages affecting commercial poultry in South America. GI-1 is a lineage deriving from the live vaccine strain Massachusetts largely used in immunization programs in all of South America, GI-16 is more widespread in Western countries bordering the Pacific Ocean (Chile, Colombia, Peru, etc.) [24,33], and GI-11 is more frequent in Brazil, which borders the Atlantic Ocean [3,34]. Noteworthily, IBV lineages’ diversity in South America follows a similar pattern to that of IBDV variants. Although this study did not assess the connections between countries, viral migration appears to be highly dependent on the different commercial relationships among western and eastern countries in South America. Bridges between these countries need to be identified to develop more effective international control protocols to avoid IBV dispersal [6,11]. 

Another interesting result reported here was the IBV GI-23 dynamics in Brazil. Noteworthily, many poultry farms from South Brazil reported infectious bronchitis outbreaks before the first detection of IBV GI-23 [7,35]. Severe clinical manifestations in the upper respiratory tract and lesions in the kidney lesions were reported in most affected broiler flocks. At necropsy, the main findings were mucus and congestion in the tracheal mucosa [35]. As a consequence, a significant increase in the mortality rate was observed as well as condemnations at slaughterhouses [7,35]. All these aspects taken together alarmed poultry companies and veterinarians, who gradually started to adopt stricter biosecurity measures. This sequential history also seems to have been confirmed in the phylodynamic analysis, where the IBV GI-23 population in Brazil increased progressively in 2021 before proper diagnostics could be employed. From the moment that IBV GI-23 was recognized as the cause of the recent outbreaks of infectious bronchitis by the large poultry farming industries (around December 2021), they began to apply more intensive vaccination protocols and adopt stricter biosafety measures. These efforts to control IBV GI-23 could explain the reduction in the viral population in the first months of 2022 followed by a later stabilization at a lower level, avoiding an even wider spread in Brazil. The capability of IBV GI-23 to extend its geographical range reinforces the need for an even more comprehensive investigation of the phylodynamic evolution of this lineage worldwide [11]. 

In the analysis of the S1 amino acid sequence alignment, all 120 Brazilian IBV GI-23 strains could be clearly divided into the same two groups (SA.1 and SA.2) with specific molecular signatures. All these amino acid substitutions occurred in the HVR1/2; many of them were associated with epitopes in the S1 protein and relevant for chicken cell receptor binding function and immune protection [36,37]. Importantly, vaccination programs with homologous and heterologous IBV strains have been implemented in an attempt to control Variant 2 in poultry farms from Africa, Asia and Europe [8,38]. More studies are necessary to evaluate the performance of traditional/novel vaccines and immunization programs adopted to control these highly disseminated IBV GI-23 subclades (VOCs, “variants of concern”) in Brazil/South America. 

## 5. Conclusions

In conclusion, the present study demonstrates the recent introduction (~2017 to 2019) of IBV GI-23 in Brazil and South America. It also suggests Brazilian IBV GI-23 strains originated from Eastern Europe, a continent region with a high recent spread of this lineage. Four IBV lineages (GI-1, GI-11, GI-16 and GI-23) are now spread throughout South America. Continuous epidemiological surveillance for them is necessary in poultry farms. Novel studies are also necessary to define better strategies for IBV GI-23 prevention and control in the South American poultry industry. 

## Data Availability

The data presented in this study are openly available in GenBank with the numbers OQ884498–OQ884591.

## Supplementary Materials

The following supporting information can be downloaded at: https://www.mdpi.com/article/10.3390/v15061229/s1, Figure S1: Maximum clade credibility tree constructed using 200-S1 subunit complete gene sequences, rooted using BEAST. Tip label colors denote the geographic origin of isolates (green for Africa, red for Asia, blue for Europe and purple for South America). Time by year is plotted along the x-axis. Branch labels denote posterior probabilities of branch support; Table S1: Metadata of 94 IBV GI-23 Brazilian isolates sequenced in this study; Table S2: Metadata 232-S1 subunit complete gene sequences [6,11]; Table S3: Metadata 389-HVR1/2 worldly sequences [6,7,11]; Table S4: Metadata for 120 HVR1/2 Brazilian sequences [11].
