# Supplementary material for: Evolution and Epidemic Spread of the Avian Infectious Bronchitis Virus (IBV) GI-23 in Brazil
Source: Viruses. 2023 May 24;15(6):1229. doi: 10.3390/v15061229 (PMC10301962; doi:10.3390/v15061229)
Supplement: Supplementary file 1 [file viruses-15-01229-s001.zip › viruses-2405015-supplementary Table S1.pdf]

Table S1. Metadata of 94 IBV GI-23 isolates sequenced in this study.

GenBank	Isolate	Country	Year
OQ884498	SB001_oct21	Brazil	2021
OQ884499	SB002_oct21	Brazil	2021
OQ884500	SB003_oct21	Brazil	2021
OQ884501	SB004_nov21	Brazil	2021
OQ884502	SB005_nov21	Brazil	2021
OQ884503	SB006_dec21	Brazil	2021
OQ884504	SB007_dec21	Brazil	2021
OQ884505	SB008_jan22	Brazil	2022
OQ884506	SB009_jan22	Brazil	2022
OQ884507	SB010_jan22	Brazil	2022
OQ884508	SB011_jan22	Brazil	2022
OQ884509	SB012_jan22	Brazil	2022
OQ884510	SB013_jan22	Brazil	2022
OQ884511	SB014_jan22	Brazil	2022
OQ884512	SB015_jan22	Brazil	2022
OQ884513	SB016_jan22	Brazil	2022
OQ884514	SB017_jan22	Brazil	2022
OQ884515	SB018_jan22	Brazil	2022
OQ884516	SB019_jan22	Brazil	2022
OQ884517	SB020_jan22	Brazil	2022
OQ884518	SB021_jan22	Brazil	2022
OQ884519	SB022_jan22	Brazil	2022
OQ884520	SB023_jan22	Brazil	2022
OQ884521	SB024_jan22	Brazil	2022
OQ884522	SB025_jan22	Brazil	2022
OQ884523	SB026_jan22	Brazil	2022
OQ884524	SB027_jan22	Brazil	2022
OQ884525	SB028_jan22	Brazil	2022
OQ884526	SB029_jan22	Brazil	2022
OQ884527	SB030_jan22	Brazil	2022
OQ884528	SB031_jan22	Brazil	2022
OQ884529	SB032_jan22	Brazil	2022
OQ884530	SB033_jan22	Brazil	2022
OQ884531	SB034_jan22	Brazil	2022
OQ884532	SB035_jan22	Brazil	2022
OQ884533	SB036_jan22	Brazil	2022
OQ884534	SB037_jan22	Brazil	2022
OQ884535	SB038_jan22	Brazil	2022
OQ884536	SB039_feb22	Brazil	2022
OQ884537	SB040_feb22	Brazil	2022
OQ884538	SB041_feb22	Brazil	2022

OQ884539	SB042_feb22	Brazil	2022
OQ884540	SB043_feb22	Brazil	2022
OQ884541	SB044_feb22	Brazil	2022
OQ884542	SB045_feb22	Brazil	2022
OQ884543	SB046_feb22	Brazil	2022
OQ884544	SB047_apr22	Brazil	2022
OQ884545	SB048_sep22	Brazil	2022
OQ884546	SB049_sep22	Brazil	2022
OQ884547	SB050_sep22	Brazil	2022
OQ884548	SB051_oct22	Brazil	2022
OQ884549	SB052_oct22	Brazil	2022
OQ884550	SB053_oct22	Brazil	2022
OQ884551	SB054_nov22	Brazil	2022
OQ884552	SB055_nov22	Brazil	2022
OQ884553	SB056_nov22	Brazil	2022
OQ884554	SB057_nov22	Brazil	2022
OQ884555	SB058_nov22	Brazil	2022
OQ884556	SB059_nov22	Brazil	2022
OQ884557	SB060_nov22	Brazil	2022
OQ884558	SB061_nov22	Brazil	2022
OQ884559	SB062_dec22	Brazil	2022
OQ884560	SB063_dec22	Brazil	2022
OQ884561	SB064_dec22	Brazil	2022
OQ884562	SB065_dec22	Brazil	2022
OQ884563	SB066_dec22	Brazil	2022
OQ884564	SB067_dec22	Brazil	2022
OQ884565	SB068_dec22	Brazil	2022
OQ884566	SB069_dec22	Brazil	2022
OQ884567	SB070_dec22	Brazil	2022
OQ884568	SB071_dec22	Brazil	2022
OQ884569	SB072_dec22	Brazil	2022
OQ884570	SB073_dec22	Brazil	2022
OQ884571	SB074_dec22	Brazil	2022
OQ884572	SB075_dec22	Brazil	2022
OQ884573	SB076_dec22	Brazil	2022
OQ884574	SB077_jan23	Brazil	2023
OQ884575	SB078_jan23	Brazil	2023
OQ884576	SB079_jan23	Brazil	2023
OQ884577	SB080_jan23	Brazil	2023
OQ884578	SB081_jan23	Brazil	2023
OQ884579	SB082_jan23	Brazil	2023
OQ884580	SB083_jan23	Brazil	2023
OQ884581	SB084_jan23	Brazil	2023

OQ884582	SB085_jan23	Brazil	2023
OQ884583	SB086_jan23	Brazil	2023
OQ884584	SB087_jan23	Brazil	2023
OQ884585	SB088_jan23	Brazil	2023
OQ884586	SB089_jan23	Brazil	2023
OQ884587	SB090_jan23	Brazil	2023
OQ884588	SB091_jan23	Brazil	2023
OQ884589	SB092_jan23	Brazil	2023
OQ884590	SB093_jan23	Brazil	2023
OQ884591	SB094_jan23	Brazil	2023
