# Supplementary figures and images for: Evolution and Epidemic Spread of the Avian Infectious Bronchitis Virus (IBV) GI-23 in Brazil
Source: Viruses. 2023 May 24;15(6):1229. doi: 10.3390/v15061229 (PMC10301962; doi:10.3390/v15061229)

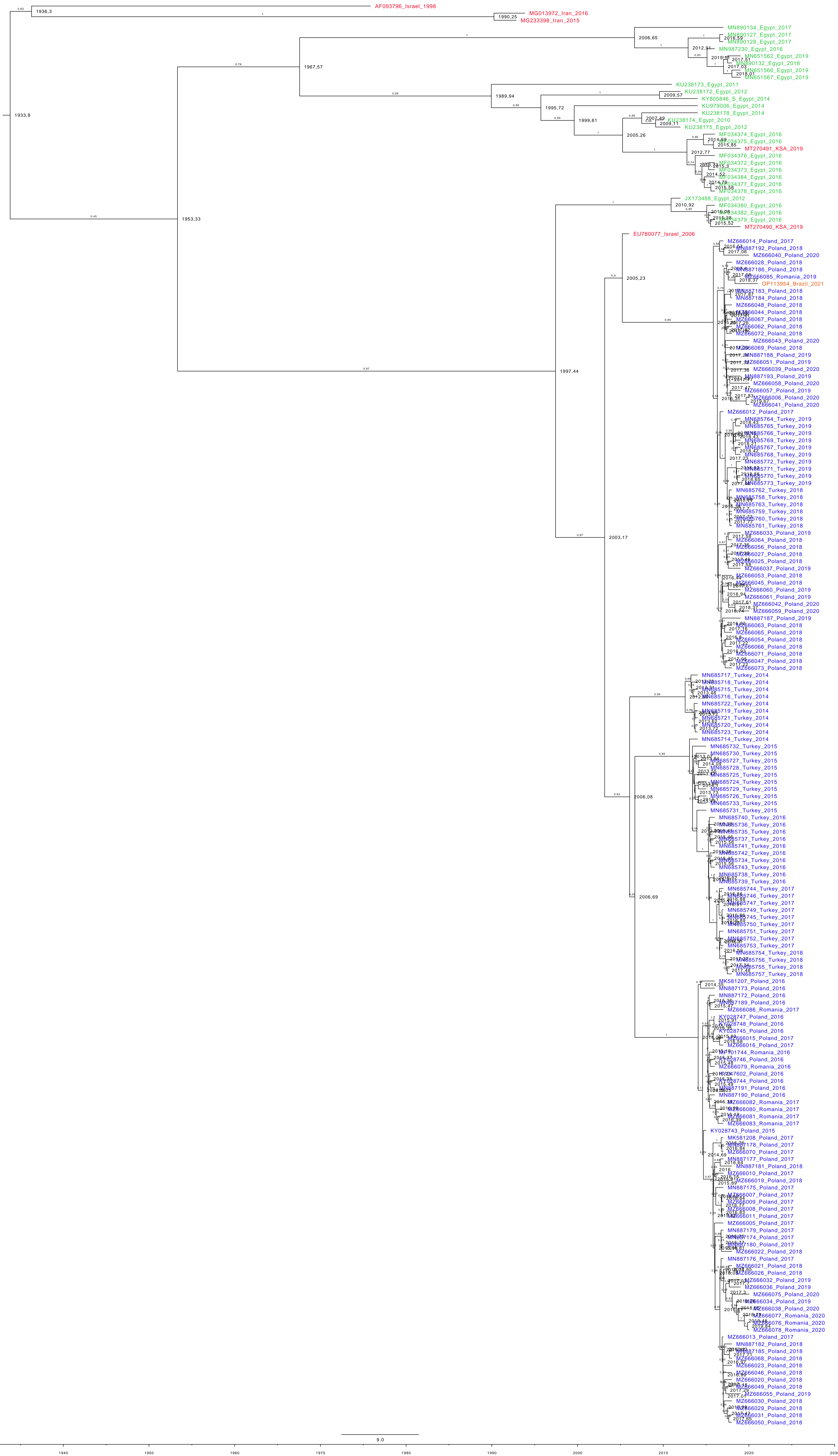

Supplement: Supplementary file 1 [file viruses-15-01229-s001.zip › Figure S1.pdf]
